# Characterization of the Use of Low Frequency Ultrasonic Guided Waves to Detect Fouling Deposition in Pipelines

**DOI:** 10.3390/s18072122

**Published:** 2018-07-02

**Authors:** Habiba Lais, Premesh S. Lowe, Tat-Hean Gan, Luiz C. Wrobel, Jamil Kanfoud

**Affiliations:** Brunel University London, Kingston Lane, Uxbridge, Middlesex UB8 3PH, UK; habiba.lais@brunel.ac.uk (H.L.); shehan.lowe@brunel.ac.uk (P.S.L.); luiz.wrobel@brunel.ac.uk (L.C.W.); jamil.kanfoud@brunel.ac.uk (J.K.)

**Keywords:** COMSOL, ultrasonic guided waves, numerical modelling, disperse, fouling detection

## Abstract

The accumulation of fouling within a structure is a well-known and costly problem across many industries. The build-up is dependent on the environmental conditions surrounding the fouled structure. Many attempts have been made to detect fouling accumulation in critical engineering structures and to optimize the application of power ultrasonic fouling removal procedures, i.e., flow monitoring, ultrasonic guided waves and thermal imaging. In recent years, the use of ultrasonic guided waves has been identified as a promising technology to detect fouling deposition/growth. This technology also has the capability to assess structural health; an added value to the industry. The use of ultrasonic guided waves for structural health monitoring is established but fouling detection using ultrasonic guided waves is still in its infancy. The present study focuses on the characterization of fouling detection using ultrasonic guided waves. A 6.2-m long 6-inch schedule 40 carbon steel pipe has been used to study the effect of (Calcite) fouling on ultrasonic guided wave propagation within the structure. Parameters considered include frequency selection, number of cycles and dispersion at incremental fouling thickness. According to the studied conditions, a 0.5 dB/m drop in signal amplitude occurs for a fouling deposition of 1 mm. The findings demonstrate the potential to detect fouling build-up in lengthy pipes and to quantify its thickness by the reduction in amplitude found from further numerical investigation. This variable can be exploited to optimize the power ultrasonic fouling removal procedure.

## 1. Introduction

Fouling formation is a major problem for many industries including the offshore industry [1]. It is an important factor contributing to the assessment of service lifetime and the safety of marine facilities [2]. Large sums of money are spent on cleaning and preventative measures to maintain offshore structures in a state of operation and efficiency. Current fouling removal methods include hydraulic, chemical and manual processes. The most common fouling mechanisms in offshore structures are the deposition of hard-scale and growth of marine organisms, accumulating in engineering structures such as pipes and ship hulls. The type of fouling is dependent on the environmental conditions surrounding the structure. Current removal methods can be costly and time consuming due to necessary halts in production. One successful method of fouling removal is the use of chemicals [3]. This achieves up to 100% de-fouling but has the disadvantage of a negative environmental impact due to the release of chemicals after use, as well as requiring down-time of the facility. Another promising method that has recently emerged is the use of ultrasound. Currently, ultrasonic baths are used for cleaning specific, individual parts of the offshore plant by generating cavitation bubbles which implode on the fouled surface [4,5], particularly in reverse osmosis applications [6,7,8]. Conventionally, fouled components are submerged into an ultrasonic bath which again requires halting operation of the structure.

Monitoring of the fouling accumulation in pipelines allows optimization of fouling removal procedures and also the detection of fouling which could affect the quality of the fluid being carried past the contaminated walls. Various methods for the detection of fouling have been discussed [9], for example, observing changes in hydrodynamics [10] and detecting changes to heat transfer parameters in the build-up of fouling [11]. Withers [12] also investigated various methods including electrical and optical processes, and discussed the acoustic methods covering pulse-echo and transmission techniques. Current acoustic methods have the advantage of detecting fouling accumulation non-invasively and, potentially, over large distances from a single test location [13,14].

This paper is organized as follows. The theoretical background of Ultrasonic Guided Waves (UWG) is given in Section 2, while Section 3 consists of the laboratory experiments. Finite Element Analysis (FEA) follows in Section 4. Numerical results are discussed in Section 5, and conclusions and future suggested work follow in Section 6.

## 2. Theoretical Background

### 2.1. Fundamentals of Ultrasonic Guided Waves

Compared to conventional Ultrasonic Testing (UT), UGW is an emerging technique and requires understanding of the elastic wave propagation within the structural boundaries to obtain a reliable assessment of the structural health [15]. Navier’s equation of motion for an isotropic elastic unbounded media is as follows (refer to Equation (1)):

(1) $$\;\left(\lambda +\mu \right)\nabla \nabla .u+\mu \nabla^{2}u=\rho \left(\frac{\partial^{2}u}{\partial t^{2}}\right)$$

where *λ* and *µ* are Lamé constants, *u* is the three-dimensional displacement vector, ∇ is the three-dimensional Laplace operator and *ρ* is the material density. Using Helmholtz decomposition, substituting into Navier’s equation, gives the following Equations (2) and (3), where $c_{l}$ and $c_{s}$ are the velocities of longitudinal and shear waves respectively.

(2) $$c_{l}=\sqrt{\frac{\lambda +2\mu }{\rho }}$$

(3) $$c_{s}=\sqrt{\frac{\mu }{\rho }\;}$$

In the derivation, there are two types of elastic waves that can propagate in solids (longitudinal waves and shear waves). These can travel in any direction. To identify the different wave modes, nomenclature for guided waves has been introduced by Silk and Bainton [16]. The vibration modes in a cylindrical structure can be denoted as follow:

(4) X(n,m)

where *X* is the vibration mode (torsional, longitudinal, flexural), *n* is an index identifying the harmonic variants of displacement around the circumference, and *m* is an index identifying the vibration complexity within the wall of the pipe. 

**Figure 1:**
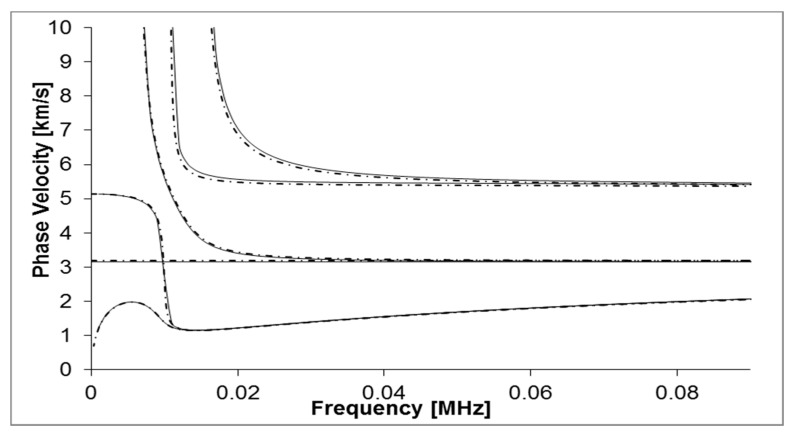
Dispersion curve for 6-inch schedule 40 carbon steel pipe cross section using DISPERSE software (solid lines) and GUIGUW software (dashed lines).

The variation in wave velocity relative to the operating frequency is known as dispersion [15]. This causes spreading of the signal when propagating through a structure, which is an undesirable phenomenon when using UGW inspection as it makes the data interpretation complex. Pavlakovic et al. [17] developed the commercial software DISPERSE which has been used to generate the dispersion curves for the structure under investigation in this study (6-inch schedule 40 carbon steel pipes as illustrated in Figure 1). The solid lines represent the computed DISPERSE dispersion curves.

Another promising software code for plotting dispersion curves is the open-source core code based on Semi Analytical Finite Element methods (SAFE) known as GUIGUW (Graphical User Interface for Guided Ultrasonic Waves) [18]. This code has been developed in MATLAB and is a stand-alone software. Its advantages are; enhanced numerical stability, computational efficiency and it allows multiple layers to be investigated. For the current paper, it is useful to investigate the effects of the addition of a fouling layer when generating dispersion curves. Figure 1 displays the dispersion curves generated by the GUIGUW software as dashed lines. The graph shows reasonable agreement between both dispersion plotting codes.

### 2.2. Current State of the Art of Ultrasonic Guided Waves

The commercialization of Ultrasonic Guided Wave (UGW) systems began in the late 1990s. Current commercial UGW systems are listed in Table 1 [19]. Dependent on the UGW system, the systems can inspect pipelines that are coated, insulated, buried or operating at high temperatures. UGW systems can inspect not only pipelines but also tanks, bridges and offshore structures. Primarily, the method has been used to detect anomalies in engineering assets where they can lead to catastrophic bursts and failures. There has been recent work on applying the UGW technique for fouling detection [9], specifically for food industry applications carrying food/liquids. Lohr & Rose [14] used a 2.62 MHz piezoelectric transducer on an angled Plexiglas wedge to produce the non-leaky longitudinal wave S0 through a stainless steel pipe. The results showed that the amplitude decreases with the addition of the fouling layer (tar) seen in the L(0,5) mode with an increase in fouling thickness. Hay & Rose [13] also investigated the use of Ultrasonic Guided Waves for fouling detection using a comb sensor operating at 2.5 MHz attached to a stainless steel pipe. The longitudinal mode L(0,4) showed high sensitivity to the addition of fouling. Both investigations [13,14] operated at a higher frequency range (MHz) and also only studied longitudinal waves; this limits the length fouling detection from one location due to the higher level of attenuation. The current study focuses on the use of a lower frequency range (kHz) and torsional wave modes to achieve a prolonged coverage using a single location.

UGW was also used to detect fouling in a duct using the acoustic hammer technique [20,21,22] and an ultrasonic transducer wedge at 500 kHz [21]; however, the research focused on signal processing aspects of the received signal to detect fouling. The application of acoustic hammer is inadvisable for industry use as the hammer impact may be inconsistent and may vary in amplitude, resulting in difficulties of comparison between the amplitude changes due to the accumulation of fouling and those due to the impact of the hammer itself. The transducer wedge application used was operated at a high frequency and did not specify the wave mode used in the investigation.

The UGW research on fouling detection has shown it to be sensitive to the change in material and thickness of layers [9]. The method itself is non-invasive and can be used whilst fouling removal is being carried out to monitor the cleaning. Another area that has not been investigated is the application of UGW to long range fouling detection. Recent investigations have focused on smaller samples which may justify why longitudinal mode excitation has been used [13,14] as it is dispersive but it is only being applied to a short specimen due to the excitation at MHz range [15]. The low frequency UGW has been used to inspect tens of meters of pipes for over two decades (commercial system i.e., Teletest [23]) due its low attenuation as an inherent characteristic; furthermore, benefits of using low frequency UGW over conventional UT have been reported in the literature for the inspection of elongated structures [24]. The current study investigates the use of fundamental torsional mode T(0,1) for its non-dispersive characteristics over the operating frequency range of UGW (20–100 kHz) for long-range detection.

### 2.3. Finite Element Analysis

A review of numerical modelling methods has been discussed in depth by Wallhäußer et al. [9], where various research studies have attempted to model and predict fouling. The benefits of modelling are the ability to predict the amplitude drop, attenuation and other parameters to relate these to the presence of fouling. This allows the development of fouling to be predicted and removed before the structure reaches a detrimental condition that results in pipe blockage, bursts and human casualties. More specifically, predictive models can be used for comparison when monitoring real-time data from a structure. This collective data can be cross-referenced with the predictive model to determine the extent of fouling build-up. 

The SAFE method is commonly used for generating dispersion curves for different structures. An example of this is the modelling of hollow cylinders with coatings to generate dispersion curves and attenuation characteristics of axisymmetric and flexural modes [25]. 3D hybrid models have also been investigated, combining both SAFE and Finite Element Analysis (FEA), to model UGW interaction with non-axisymmetric cracks in elastic cylinders [26], allowing the technique to be used on defects with complex shapes.

FEA methods have been applied to model UGW propagation within a structure for more specific applications. For example, a 2D FE model was used for UGW propagation in complex geometries and proved to be more effective than analytical solutions [27]. UGW propagation in bones has been modelled using FEA of the fracture callus and healing course within a three-stage process [28]. 3D numerical simulations have been carried out on UGW for non-destructive inspection of CFRP rods with delamination [29].

The software code ABAQUS has been used to model UGW propagation for long-range defect detection in rail road tracks [30]. ABAQUS has also been used to model longitudinal and torsional wave propagation on a cylinder [31]. The optimal excitation mode was selected using signal processing algorithms and used the reflection coefficient for defect sizing. Although ABAQUS can successfully model UGW propagation, COMSOL Multiphysics has recently become more popular due to its multiphysics and post-processing capabilities. For example, COMSOL has been used to model UGW propagation in the frequency domain, later converted to the time domain using a Fast-Fourier Transform (FFT) [32] which reduces computational time.

## 3. Laboratory Experiment and Results

### 3.1. UGW Inspection

**Figure 2:**
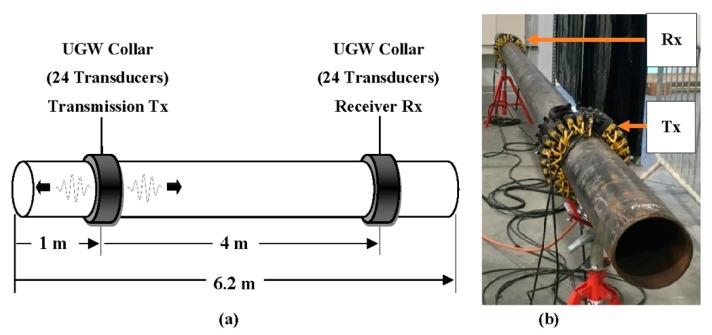
(**a**) Schematic and (**b**) set-up of the UGW collar arrangement for the current investigation on a 6.2-m 6-inch schedule 40 carbon steel pipe for data collection using pitch-catch configuration.

Laboratory experiments were conducted to investigate the UGW propagation within a 6.2-m long 6-inch schedule 40 carbon steel pipe. This study was conducted to characterize the change in UGW propagation as an effect of the presence of fouling within the pipe wall. Two Teletest^®^ UGW collars were used to collect data in pitch-catch configuration to ease the data interpretation. Each collar consists of 24 transducers evenly spaced around the circumference of the 6-inch schedule 40 carbon steel pipe. The transmission collar is placed 1 m away from the pipe end and the receiving collar is placed 4 m away from the transmission collar as shown in Figure 2. A tool lead is connected to both collars to synchronize the data collection. Baseline data are collected from the clean pipe before generating hard-scale fouling on the inner wall of the pipe of the type known as Calcite. Data collection is implemented by transmitting a torsional wave mode from the transmission collar and monitoring the transmitted signal from the receiving collar. The data collection was performed over a frequency range of 30–80 kHz in 1 kHz increments. Furthermore, different number of cycles for the input signal was also considered to state the optimum number to use in order to detect fouling higher sensitivity.

The excitation signal applied is a sine wave modulated using the Hann window function (refer Equation (5)).

(5) $$U\left(t\right)=\frac{1}{2}\sin\left(2\pi ft\right)\left[1-\cos\left(\frac{2\pi ft}{n}\right)\right]$$

where *t* is time, *f* is the central frequency and *n* is the number of cycles.

### 3.2. Fouling Generation

**Figure 3:**
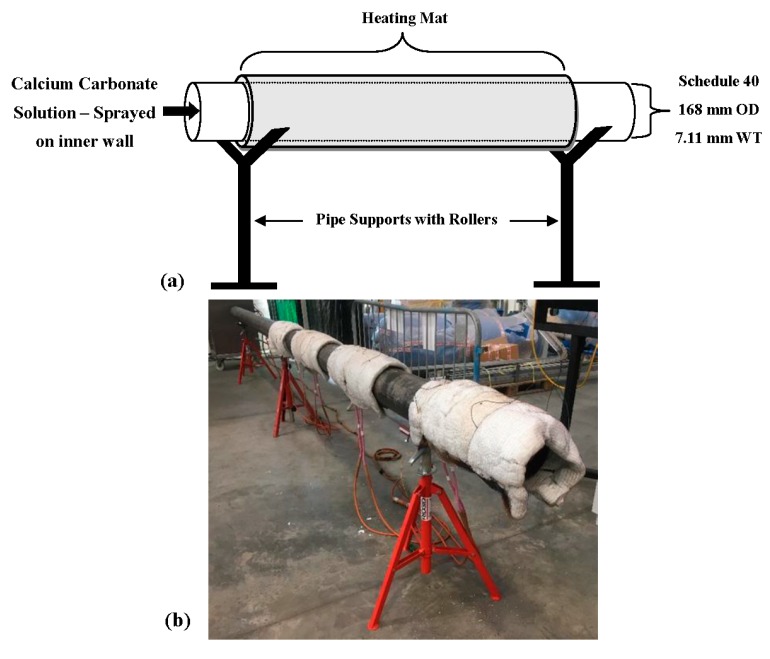
Fouling generation of 6.2-m 6-inch schedule 40 carbon steel pipe (**a**) schematic and (**b**) experimental set-up.

To obtain data for comparison with the COMSOL model, fouling was generated on the inner wall of a pipe by heating the outer wall of the pipe up to 120 °C whilst spraying a highly concentrated calcium carbonate solution on the inner wall as illustrated in Figure 3. A Cooper heating system [33] was used to heat four heating mats wrapped around the pipe, and further wrapped with carbon fiber insulation as shown in Figure 3. Three thermocouples were placed between each mat to monitor the temperature to allow the Cooper heating system to reach and maintain the target temperature. Fifty liters of deionized water solution were prepared with 1.5 g/L of Calcium Chloride and 1.5 g/L of Sodium Bicarbonate. The highly concentrated mixture was placed into a manual pressure sprayer connected to a 3.2-m telescopic pressure sprayer lance. 

**Figure 4:**
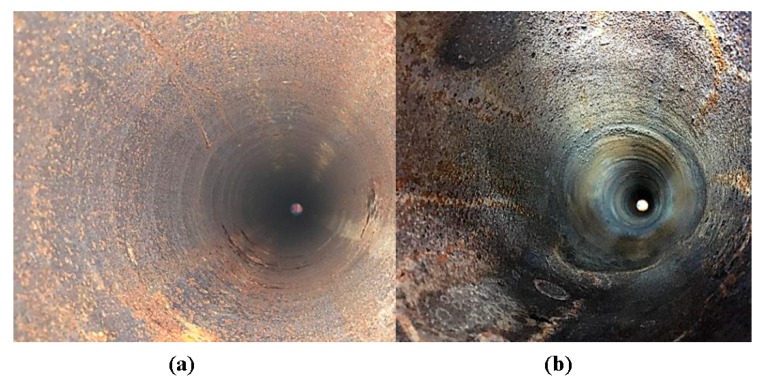
Image of the inner wall of the 6.2-m 6-inch schedule 40 carbon steel pipe (**a**) displaying some corrosion before commencing fouling generation procedure and (**b**) after generating a layer of Calcite.

Figure 4a displays the inner pipe wall before undergoing fouling. Figure 4b shows successful generation of hard-scale fouling. There is some corrosion on the inner walls. The fouling generation is carried out and achieves a layer of Calcite on the inner pipe wall. After creating a layer of fouling on the inner pipe wall, the UGW Teletest collars were placed at their original locations to collect further data for analysis.

### 3.3. Experimental Results

**Figure 5:**
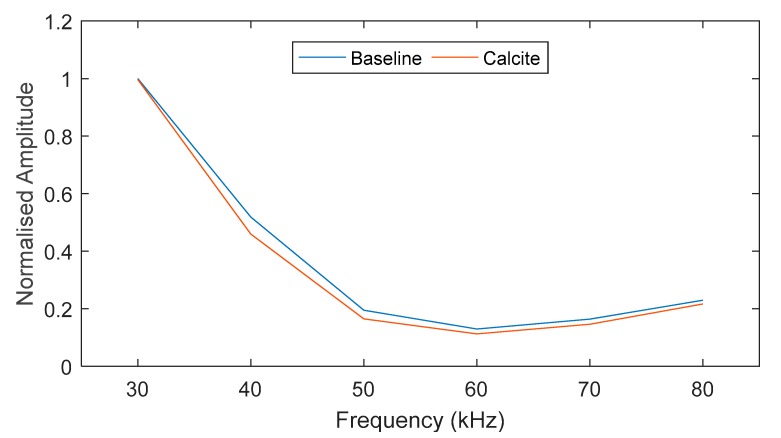
Comparison of maximum receiving amplitude at different input frequencies.

The laboratory experiment was conducted over a frequency range of 30–80 kHz. The maximum amplitude of the monitored pulse is plotted in Figure 5. For the studied case, there is an 80% drop in amplitude at 50–80 kHz and, therefore, this frequency range was neglected in this study (marked in black dashed line). Based on the results, the frequency range of interest for this study is 30–45 kHz. There was a reduction in sensitivity for the Calcite layer at the lower end of the frequency range (<40 kHz) due to having a comparatively larger wavelength. Therefore, 45 kHz was selected in this study for further analysis. 

**Figure 6:**
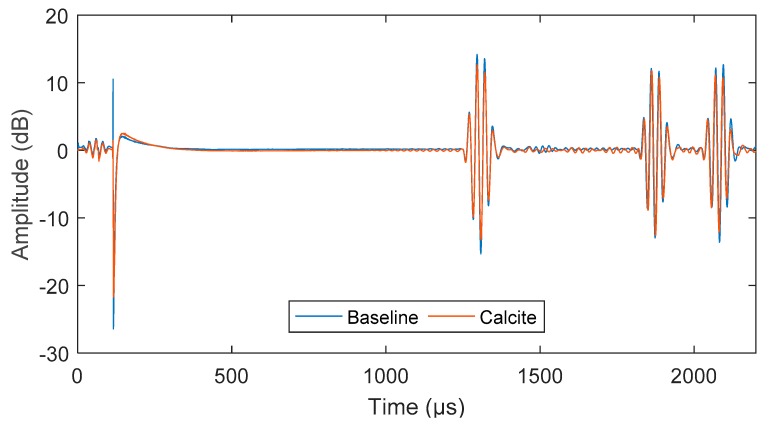
Received UGW signal for baseline and Calcite, displaying drop in amplitude.

**Figure 7:**
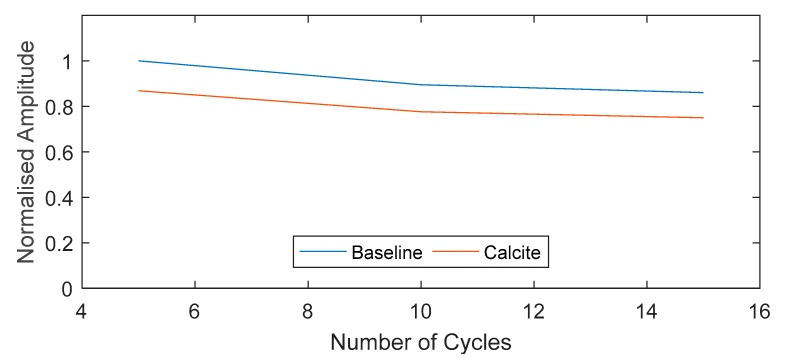
Experimental results—Comparison of maximum amplitude for baseline and Calcite signal at different number of signal cycles.

The signal obtained from exciting a 5-cycle torsional mode at 45 kHz was compared for both baseline and fouled pipe in Figure 6. There is a 2 dB drop in amplitude over 4 m (0.5 dB/m) due to the presence of fouling. The number of cycles is also compared in Figure 7, which shows approximately a 20% drop in signal amplitude at 5, 10 and 15 cycles.

The frequency bandwidth of the input signal can be calculated as follow:

(6) $$F_{bw}=f\pm \;\frac{\left(k_{i}+2\right)f}{n}$$

where *F_bw_* is the frequency bandwidth, *k_i_* is the bandwidth of the desired lobe (where *k_i_* = 0 for main lobe and *k_i_* = 1 is for the first side lobe).

At a particular frequency, the bandwidth of the excited pulse is dependent on the number of cycles [34]. The frequency bandwidth (main lobe) of the 5-cycle input signal is in the frequency range of 27-63 kHz whereas the bandwidth (main lobe) of the 15-cycle input signal is 39–51 kHz. As shown in Figure 5, there is a higher amplitude response when the frequency gets lower due to having a larger wavelength. This behavior is asymptotic but the amplitude variation over different number cycles is low and can be negligible due to the low attenuation and non-dispersive characteristics of T(0,1) mode. However, this behavior can be detrimental for the excitation of longitudinal modes.

## 4. Numerical Investigation

To aid understanding of the wave propagation over a pipeline with and without fouling accumulation, an FEA model was created in COMSOL Multiphysics 5.3. The model followed the geometry of the 6.2-m, 6-inch schedule 40 carbon steel pipe and replicated the geometry and placement of the transmission and receiving transducers for pitch-catch configuration for transmitting and receiving the signal data, as shown in Figure 2a.

Transmission points were placed at 1 m from one end of the pipe to simulate 24 transducers in the experiment. For ease of computation, symmetry was invoked to analyse 1/48th of the complete model permitting just one point load to be applied. The point load was applied in a direction dependent on the wave mode being excited—for a torsional mode, this is applied perpendicular to the length of pipe. The pressure point load is a 5-cycle sine wave modulated using the Hann window function (refer to Equation (5)). The receiving point is placed 4 m away from the transmission point.

A dynamic transient simulation to map out the propagation of the wave requires the calculated mesh to be optimal. The wave equation requires the time stepping within the solver to complement the meshing itself to yield an accurate solution. The meshing size requires a minimum of 8 2nd-order mesh elements per wavelength. The equation used to calculate the maximum allowed element size (*h_o_*) [14,35]:

(7) $$h_{o}=\frac{c}{Nf_{o}}$$

where *c* is the velocity, *N* is the number of elements per wavelength and *f_0_* is the center frequency.

The fouling model was created in the same manner. However, a 1-mm solid layer was modelled on the inner wall of the pipe to represent the expected thickness of fouling to attach to the pipe during experimentation. The properties of this layer can be found in Table 2.

The COMSOL model investigated the torsional mode over 30–45 kHz in 5 kHz steps. After selecting a frequency, this model was used to investigate the addition of a Calcite layer at 1-mm, 3-mm and 5-mm thickness. To validate the model, the Time of Arrival was calculated using the group velocity of the torsional mode found in Figure 1. Time of Arrival can be calculated as follows (refer Equation (8)) [35]:

(8) $$Time\;of\;Arrival=\frac{x}{c_{t}}$$

where *x* is the distance from transmitter to receiver and *c_t_* is the group velocity of the torsional wave mode at the operating frequency. 

## 5. Numerical Results and Discussions

The fouling detection experiment was conducted using the Teletest^®^ on a 6.2-m 6-inch diameter schedule 40 carbon steel pipe (baseline and fouled). Experimental results were compared to the FEA results to achieve a good correlation with the effects of the additional fouling layer.

**Figure 8:**
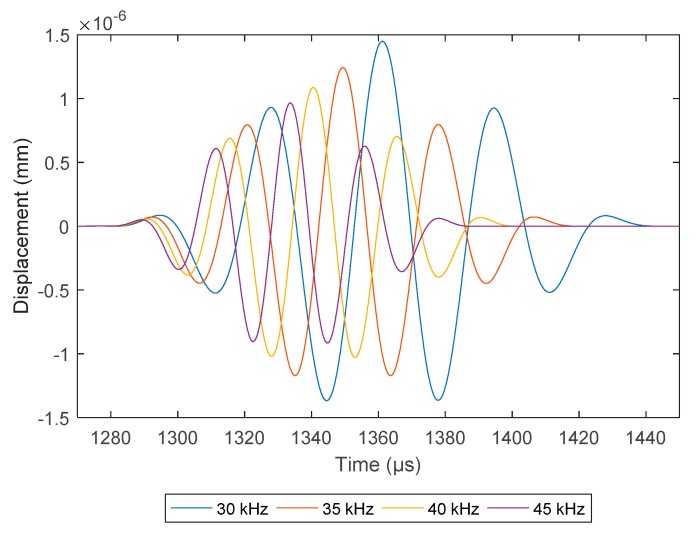
Comparison of FEA pitch-catch signal at different operating frequencies.

**Figure 9:**
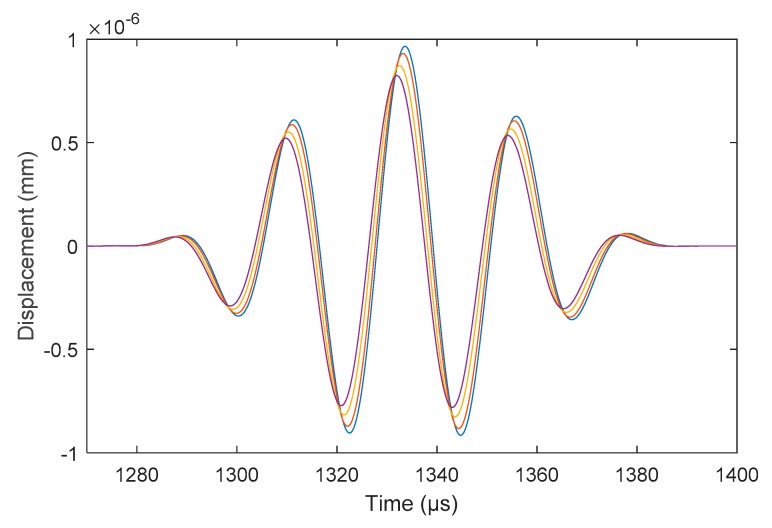
Comparison of FEA pitch-catch signal at different fouling thicknesses.

The COMSOL model investigated 30–45 kHz signals at 5 kHz increments. 45 kHz was selected due to the signal having a shorter pulse length as shown in Figure 8. At this frequency, the addition of the Calcite layer was investigated with 1-, 3- and 5-mm of fouling thickness on the inner pipe wall. Compared to the baseline model in Figure 9, the receiving pulse shows a drop in amplitude with the increase of the Calcite layer thickness.

There is a shift in Time of Arrival in Figure 9 with the incremental thickness of the Calcite layer. With the increase in Calcite thickness, the pulse of interest arrives faster, this is potentially due to the change in the velocity of the T(0,1) mode with the incremental Calcite thickness. The GUIGUW [18] code was used to plot the dispersion curves against the incremental fouling layer and is tabulated in Table 3. Adding a fouling layer to the pipe increases the velocity of the torsional mode. Although the increase is small, this finding can be concluded as the cause of the shift in Time of Arrival. Using the velocity found for each thickness of Calcite, the Time of Arrival can be calculated (refer Table 3). The Time of Arrival is calculated using the peak-to-peak values of the signal. There is a 1% error in the comparison of theoretical and numerical Time of Arrival. When comparing the variable difference between the Time of Arrival for each case, it is immaterial due to focusing on small thicknesses of Calcite which would make the data harder to use for interpreting the fouling thickness in comparison to the amplitude drop.

## 6. Conclusions and Future Works

The present paper investigates the capability of using Ultrasonic Guided Waves for detection of hard-scale fouling in pipelines. A 6.2-m long 6-inch schedule 40 carbon steel pipe was used in this investigation. For comparison, the pipe underwent fouling generation treatment at an increased rate prior to the data acquisition which shows a 0.5 dB/m drop in signal for the addition of a 1-mm thick Calcite layer. An experimentally validated FEA was used to study the effect on UGW propagation at incremental thickness of fouling. With increase in thickness, the amplitude of the signal was shown by the largest reduction in amplitude shown by the 5-mm Calcite case. The shift in Time of Arrival due to the fouling thickness has been discussed but the shift in Time of Arrival is low and this would be unsuitable for characterizing the fouling thickness due to the required sensitivity and other features that may build up within a pipe wall that can affect this small shift in time such as corrosion. This work demonstrates the potential of using UGW for long range fouling detection in pipeline based on the 0.5 dB/m amplitude reduction due to a 1-mm Calcite layer. It is further numerically investigated that the amplitude drop due to Calcite thickness can be used to characterize the thickness of the fouling due to the significant drop relative to fouling thickness. This variable can be used to support the optimization of the power ultrasonic fouling removal procedure in future work. Furthermore, this experimentally validated numerical simulation can also be used to optimize the fouling detection capabilities as part of the future developments in this technology and also the sensitivity and the level of attenuation has to be compared against the conventional UT in future studies to get a performance evaluation. 

## Figures and Tables

**Table 1:** Comparison of Ultrasonic Guided Wave systems on the commercial market.

Supplier	UGW System	Method of Excitation	Operating Frequency Range	Inspection Range
Plant Integrity Ltd. (EddyFi)	Teletest^®^ Focus+	PZT array	20–100 kHz	60 m
Guided Ultrasonic Ltd.	Wavemaker^®^ G4	PZT array	15–80 kHz	60 m
Structural Integrity Associates Inc.	PowerFocus™	PZT array	20–85 kHz	150 m
Olympus corporation	UltraWave^®^ LRT	PZT array	15–85 kHz	90 m
Innerspec Technologies Inc.	Temate^®^ MRUT	Magnetostrictive coils	0.1–1 MHz	1–5 m
NDT-Consultant Ltd.	MsSR^®^ 3030R	Magnetostrictive coils	2–250 kHz	-

**Table 2:** Assumed material property of steel and Calcite for the Finite Element Analysis.

Material Property	Carbon Steel	Calcite
Density	7830 kg/m^3^	2700 kg/m^3^
Young’s Modulus	207 GPa	70 GPa
Poisons ratio	0.33	0.3

**Table 3:** Comparison of theoretical and FEA Time of Arrival.

	Torsional Group Velocity	Theoretical Time of Arrival at 4 m (µs)	COMSOL Pk-Pk Time of Arrival (µs)	Error %
Baseline	3152.56	1268.81	1282.60	1.09
1 mm	3152.77	1268.73	1282.00	1.05
3 mm	3153.12	1268.59	1281.80	1.04
5 mm	3153.40	1268.47	1281.20	1.01
